# DRG payment, financial signals, and low-value hospitalizations in China

**DOI:** 10.3389/fpubh.2026.1797063

**Published:** 2026-05-05

**Authors:** Lixiang Wu, Ni Wu, Lihong Lin, Qin Ying, Xiaoyuan Zhou, Yangyang Zhu

**Affiliations:** 1 The Quzhou Affiliated Hospital of Wenzhou Medical University, Quzhou People’s Hospital, Quzhou, China; 2 School of Business, Sichuan University, Chengdu, China; 3 West China School of Public Health, Sichuan University/West China Fourth Hospital, Sichuan University, Chengdu, China

**Keywords:** DRG payment, health insurance, healthcare reform, hospital incentives, low-value care

## Abstract

**Objective:**

To examine whether China’s Diagnosis-Related Group (DRG) payment reform is associated with low-value inpatient admissions—defined as entire hospitalizations of questionable necessity—and to identify the primary drivers of this type of care.

**Methods:**

We developed a new rule to identify low-value admissions using claims data, synthesizing international standards and Chinese health policies. We conducted a analysis of all DRG payment records (2022–2024, N = 251,811) from a large tertiary hospital in a region that adopted the reform early. A multilevel mixed-effects logistic regression model was applied, with the DRG Medical Expense Ratio (DER)—a measure of profitability at the case level—as the key explanatory variable.

**Results:**

The overall incidence of low-value hospitalizations was 4.86% and rose by 76.5% from 2022 to 2024, resulting in insurance expenditures of 51.23 million Chinese Yuan. Admissions with the highest profit margins (low DER) were significantly more likely to be low-value (high vs. low DER: OR = 0.056). By 2024, 80.74% of low-value cases were classified as “check-up dominated” admissions.

**Conclusion:**

Within the DRG payment environment in China, the financial incentives created by the reform are associated with hospitals admitting patients for low-value, low-complexity care, effectively shifting rather than eliminating waste in the healthcare system.

## Introduction

Diagnosis-Related Group (DRG) systems are designed to offset the volume-driven incentives inherent in fee-for-service models, so that payers can provide bundled payments to healthcare providers for each inpatient case and promote medical efficiency and cost containment (1, 2). Amid the global shift toward value-based healthcare, reform of payment systems has become a pivotal policy tool. To ensure the sustainability of its universal health coverage system amidst rising medical expenditure and a rapidly aging population, China has also launched a transformative policy of the full-scale adoption of DRG payment mechanisms since 2019 (3).

This reform is predicated on a key assumption: financial incentives will drive hospitals to systematically eliminate waste, with the ideal goal of reducing low-value care—defined as medical services with uncertain clinical benefits but clear potential for harm or resource costs (4, 5). Here low-value care refers to discrete medical services that provide limited clinical benefit for a given patient, such as unnecessary antibiotic use or inappropriate CT scans and so on. In contrast, low-value hospitalization (the focus of this study) refers to entire inpatient admissions with questionable clinical necessity.

The intended incentives of DRG payment lie in its “no reimbursement for overspending, savings kept” rule, which creates two profit paths for hospitals: boosting efficiency to cut real costs, or selecting case mixes where actual costs fall below DRG payment rates. The former aligns with the reform’s value-based goal, while the latter poses a key policy risk as it is more readily actionable. However, international experience reveals a more complex reality. A substantial body of international literature has examined how DRG payment affects provider behavior. For example, recent research has shown that capitated prepayment models in Medicare Advantage and DRG-based hospital reimbursement systems can create strong incentives for strategic behaviors including cherry-picking low-cost, low-complexity cases, a pattern that aligns closely with the low-value hospitalizations observed in this study (6, 7).

For a long time, China’s healthcare system has been dominated by large tertiary hospitals and historically reliant on volume-driven revenue expansion (8). This context provides a typical research setting to observe the interactive effects of prospective payment reform incentives and hospital operational adaptation strategies—specifically, national DRG bundled payment policies and provider-side cost-control as well as case-adjustment behavioral responses (9). While the initial phase of DRG implementation has successfully established a complex technical infrastructure for grouping and pricing (10), a critical gap in the policy design remains: while the current DRG model strongly promotes “unreasonable expense control,” it lacks embedded mechanisms to ensure that savings result from genuine efficiency improvements and the reduction of ineffective services, rather than from avoiding clinically necessary but costly care or compromising quality. This flaw raises a pivotal question: Under fixed-price pressure, will hospitals systematically favor admitting patients with simple, predictable conditions yet low clinical necessity? Furthermore, might this shift in case mix ultimately undermine the healthcare system’s overall capacity to manage complex diseases and safeguard health equity?

Existing Chinese research on low-value care is in its infancy, primarily focusing on measuring specific unnecessary procedures (11). In contrast, international studies have explored the association between DRG payment and low-value admissions, including.

inappropriate hospitalizations, overuse of diagnostic testing, and cherry-picking of low-cost, low-complexity cases under prospective payment systems (12, 13), but findings are context-dependent and cannot be directly generalized to China’s unique healthcare system. To date, there remains a lack of in-depth evidence on how the DRG payment structure is linked to systematically low-value hospitalization—defined as admissions where the clinical necessity of the entire inpatient stay is questionable. This represents a distinct and understudied problem that is more impactful than the overuse of isolated low-value services.

This study addresses this gap by investigating: What is the scale, financial burden, and primary driver of low-value hospitalizations within a major Chinese tertiary hospital operating under DRG payment (implemented since 2018)? We aim to: (1) develop and validate a transparent, data-based rules for identifying low-value inpatient admissions; (2) quantify their incidence, trend, and economic impact on the insurance fund; (3) empirically test the central hypothesis that the ratio of a case’s resource consumption to the average reimbursement cost of its DRG group is the primary predictor of its likelihood of being low-value; and (4) derive specific, technically feasible policy recommendations to integrate value-based principles directly into the operational core of China’s DRG systems.

## Methods

### Study design and setting

We conducted an observational study utilizing comprehensive hospital administrative and claims data. The study was set in City Q of Eastern China that was among the first regions to implement DRG payment. The study was carried out at the city’s leading Grade A Tertiary hospital, which is a high-volume institution. It treats approximately one-third of the city’s inpatients and faces prototypical pressures and incentives under the DRG reform.

### Data sources

We analyzed the hospital’s complete universe of finalized DRG settlement claims from January 1, 2022, to December 31, 2024 (N = 251,811). Each record constituted a closed inpatient episode and included: patient demographics, insurance type, primary diagnoses (ICD-10), procedures (ICD-9-CM-3), detailed cost breakdowns, length of stay, assigned DRG group code and weight, and the total DRG reimbursement amount.

### Defining low-value hospitalization: a transparent rule

To systematically identify potentially inappropriate admissions using payment-related data, we developed a data-driven rule that extends beyond disease-specific analyses. The development process involved policy and guideline synthesis, multidisciplinary expert consensus, rule specification, and validation. First, we synthesized international low-value care initiatives (e.g., US Choosing Wisely, UK NICE “Do Not Do”) (14–19)and Chinese national/local policy documents targeting inappropriate admissions and low-indication admission (20–22). These documents consistently define low-value care as services with limited clinical benefit, high resource consumption, and viable outpatient alternatives— criteria incorporated into our rule to ensure alignment with global and national standards.

Next, a multidisciplinary panel consisting of 3 clinicians (specializing in internal medicine, general surgery, and preventive medicine), 2 medical coders (with over 10 years of DRG coding experience), 2 insurance administrators (familiar with local DRG reimbursement policies), and 1 epidemiologist (specializing in health services research) iteratively drafted and refined identification criteria.

Final rules defined three mutually exclusive categories based on diagnosis, cost structure, and length of stay (Table 1). We clarify the core differences between Category I guideline-violating low-value hospital admissions defined in our research and domestic Ambulatory Care Sensitive Conditions (ACSCs) in China. ACSCs refer to diseases for which unnecessary hospital admissions can be effectively avoided through standardized and timely outpatient treatment as well as primary healthcare management. Admissions related to ACSCs reflect deficiencies in the accessibility, capacity, and quality of grassroots medical services, and serve as a key indicator for evaluating the effectiveness of the hierarchical medical system (23). In contrast, when defining Category I guideline-violating admissions, this study focuses on the compliance and rationality of clinical admission behaviors rather than the diseases themselves. Such inappropriate admissions arise from non-standard clinical practices and regulatory loopholes, requiring targeted rectification of unreasonable inpatient behaviors to achieve refined management and control of medical insurance funds.

**Table 1:** Criteria for defining low-value hospital admissions.

Category	Conceptual definition	Operational rules in claims data
I. Guideline-violating admission	Conditions with clear outpatient management guidelines.	Primary ICD-10 code on a predefined list of ambulatory-sensitive conditions (e.g., J06.9 acute URI, K52.9 non-infective gastroenteritis) AND length of stay ≤ 2 days.
II. Check-up dominated admission	Admission primarily for diagnostic work-up, not active treatment. Such admissions have limited clinical benefit, as diagnostic services can be feasibly completed in an outpatient setting, and hospitalization imposes unnecessary costs on patients and the health insurance system.	Sum of (Laboratory Fees + Imaging Fees) / Total Hospitalization Costs > 0.8.
III. Inappropriate maintenance admission	Admission for stable chronic disease management or convalescence.	Primary diagnosis of stable chronic condition (e.g., I10 essential hypertension) OR Z-code for follow-up (Z48-Z54) AND no major therapeutic procedure (ICD-9-CM-3 code of surgical or significant interventional nature).

Meanwhile, we conducted a comparative analysis of clinical and economic indicators between three Low-value categories admissions and non-low-value hospitalizations during the study period, including average length of stay and average direct medical expense. Validation was conducted through a double-blind manual review of a random sample of 200 cases by two independent senior clinicians. The rule demonstrated high concordance with clinical judgment (Cohen’s Kappa = 0.81).

### Statistical analysis

Descriptive Analysis was conducted to calculated annual incidence, total DRG payments, and trends. This analysis helped quantify the economic burden of low-value admissions and provide additional evidence for the validity of the low-value definition.

To identify predictors while accounting for the nested structure of patients (level 1) within clinical departments (level 2), we fitted a series of two-level logistic regression models (See Supplementary Material Text S1 for the specific formulas). The outcome is binary indicator of low-value hospitalization. To capture the immediate financial incentive of DRG payment for hospitals, we constructed a core explanatory variable at the case level—the DRG Medical Expense Ratio (DER).

This variable is calculated as: Actual medical expenses of the case / Average expenses of the corresponding DRG group. Here, the numerator, actual medical expenses of the case, refers to the total actual medical expenses incurred for the inpatient case. The denominator, average expenses of the corresponding DRG group, represents the mean medical expenses across all cases in the assigned DRG group. A ratio below 1 indicates that the actual medical expenses of the case are lower than the average expenses of its DRG group, suggesting that the case involves lower resource utilization and less intensive medical services relative to the average case in the same DRG group. Under the fixed-payment DRG system, lower actual expenses for a given DRG payment translate to a higher financial surplus for the hospital. Thus, a lower DER may create a potential economic incentive for hospitals to admit patients with similar clinical profiles. This definition aligns with the classic methodological framework in health economics, which standardizes the comparison of individual case-level expenses against the average resource consumption within corresponding DRG groups for prospective payment evaluation (24).

The DER categories were established in accordance with the DRG payment policy of Zhejiang province (25), specifically defined as follows: Low DER ≤ 0.4 times; High DER refers to cases where the total inpatient expense is significantly higher than the average expense of the corresponding DRG group (specifically, ≥3times the average for DRG groups with lower resource allocation, ≥2times for medium-resource groups, and ≥1.5 times for high-resource groups); Normal DER includes all cases excluding those classified as Low DER and High DER. This approach ensures the cutoffs are meaningful and reflective of actual economic incentive differences in the study setting, as they are aligned with local DRG payment regulations rather than arbitrary thresholds.

Other Covariates included Patients’ age, gender, insurance type. Additionally, we attempted to include DRG group fixed effects to account for disease severity and case-mix heterogeneity. However, due to the high dimensionality of DRG groups (hundreds of categories), including DRG group dummy variables would lead to model non-convergence and unstable estimates. Therefore, DRG group fixed effects were not included in the final model. We tested a random-coefficient model allowing the effect of DER to vary across departments, as departmental strategy was a key interest.

Sensitivity Analyses were conducted to test the robustness of the findings. We conducted a sensitivity analysis excluding Category II (“Check-up Dominated Admission”) cases to examine whether the association between DER and low-value hospitalization was driven by the cost structure of this category. And we tested alternative cost thresholds (i.e., ≥75% and ≥85%) for defining Category II admissions to assess the stability of results under different operational definitions. All analyses were performed using Excel 2017 and Stata 16.0, with *p* < 0.05 considered significant.

## Results

### Prevalence, trajectory, and economic impact

Over the three-year study period, there were 239,563 non-low-value hospitalization cases, covering all types of DER. And 12,248 admissions were identified as low-value, corresponding to an overall incidence of 4.86 percent. A marked upward trend was observed: the incidence rose from 3.45 percent in 2022 to 6.09 percent in 2024, representing a 76.5 percent relative increase. The associated financial burden on the medical insurance fund was substantial and grew rapidly. Total DRG payments for these low-value cases amounted to 51.23 million Yuan, with annual expenditures escalating from 8.31 million Yuan in 2022 to 33.07 million Yuan in 2024. Category II (check-up-dominated) admissions mainly drove this growth, accounting for 80.74% of all low-value cases by 2024(Table 2).

**Table 2:** Annual distribution, category composition, and financial burden of low-value hospitalizations.

Year	Total admissions	Low-value cases (*n*)	Category I (%)	Category II (%)	Category III (%)	Incidence rate (%)	DRG payments (Million ¥)
2022	61,579	2,124	278(13.09)	920(43.31)	926(43.60)	3.45	8.31
2023	92,101	4,149	199(4.80)	730(17.59)	3,220(77.61)	4.5	9.86
2024	98,131	5,975	162(2.71)	4,824(80.74)	989(16.55)	6.09	33.07
Total	251,811	12,248	639(5.22)	6,474(52.86)	5,135(41.92)	4.86	51.23

**Figure 1:**
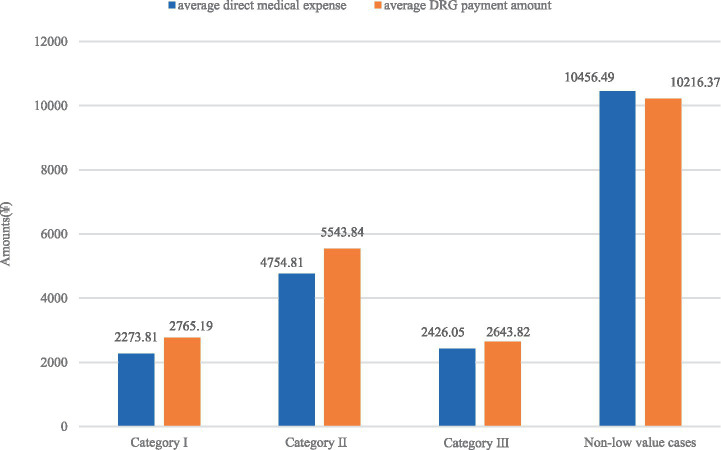
Expense and payment differences between low-value and non-low-value cases.

Validation analysis showed that all low-value admission categories (I, II, III) had significantly shorter average lengths of stay and lower economic costs compared with non-low-value cases. Specifically, the average length of stay for low-value cases ranged from 1.80 to 2.65 day, far shorter than the 5.92 days of non-low-value cases. In terms of costs, low-value cases had average direct medical expenses between 2,273.81 and 4,754.81 Yuan and average DRG payments ranging from 2,643.82 to 5,543.84 Yuan, both significantly lower than those of non-low-value cases (10,456.49 Yuan for direct medical expenses and 10,216.37 Yuan for DRG payments). Furthermore, the DRG payment-medical expense difference showed that low-value cases all had positive gaps (491.38 Yuan for Category I, 789.03 Yuan for Category II, and 217.77 Yuan for CategoryIII), indicating that DRG payments exceeded direct medical expenses, while non-low-value cases had a negative gap of −240.12 Yuan, meaning direct medical expenses surpassed DRG payments. These obvious gaps in length of stay, medical expenses, DRG payments, and payment-expense differences fully confirm the clinical and economic irrationality of low-value admissions (Figure 1).

### The dominant predictor: DRG medical expense ratio (DER)

Initial model fitting confirmed significant clustering at the departmental level (random intercept variance = 1.129, 95% CI: 0.710–1.795; likelihood ratio test χ^2^ = 4,665.50, *p* < 0.001), justifying the use of a multilevel modeling approach. The final random coefficient model demonstrated superior fit (log-likelihood = −36,460.937) compared to the random intercept model (log-likelihood = −37,149.332).

The Random Coefficient model identified DER type as the most powerful predictor.

of a low-value hospitalization (Table 3). The expense gradient was extreme. Using Low DER cases as the reference, the adjusted odds of being low-value were 65 percent lower for Normal cases (OR = 0.349, 95% CI: 0.254 ~ 0.479) and 94 percent lower for High DER cases (OR = 0.056, 95% CI:0.029 ~ 0.107). Other significant predictors included shorter length of stay and UEBMI insurance (associated with higher odds). The highly significant variance components confirmed substantial heterogeneity across clinical departments, both in the baseline risk of low-value care (σ^2^_u0_ = 1.668, *p* < 0.001) and, crucially, in the strength of the association with DER (σ^2^_vk_ = 0.968, *p* < 0.001).

**Table 3:** Results of the multilevel model.

Effects	Random intercept model			Random coefficient model		
	Odds ratio	95% confidence interval	*p*-value	Odds ratio	95% confidence interval	*p*-value
Fixed effects						
Cons	0.407	0.284 ~ 0.584	<0.001	0.401	0.239 ~ 0.669	<0.001
Year						
2023 (Ref: 2022)	0.754	0.707 ~ 0.804	<0.001	0.756	0.709 ~ 0.807	<0.001
2024 (Ref: 2022)	1.802	1.581 ~ 2.054	<0.001	1.675	1.467 ~ 1.911	<0.001
Gender (Ref: male)	1.017	0.976 ~ 1.061	0.417	1.011	0.970 ~ 1.055	0.602
Age						
18–70 (Ref: ≤18)	1.405	1.236 ~ 1.598	<0.001	1.313	1.154 ~ 1.496	<0.001
≥70 (Ref: ≤18)	1.557	1.357 ~ 1.786	<0.001	1.493	1.300 ~ 1.714	<0.001
Insurance type (Ref: UEBMI)	0.938	0.897 ~ 0.981	0.005	0.926	0.885 ~ 0.968	0.001
DER type						
Normal (Ref: low)	0.347	0.326 ~ 0.358	<0.001	0.349	0.254 ~ 0.479	<0.001
High (Ref: low)	0.104	0.089 ~ 0.121	<0.001	0.056	0.029 ~ 0.107	<0.001
Discharge outcome						
Improved (Ref: Cured)	1.024	0.784 ~ 1.337	0.861	1.218	0.937 ~ 1.582	0.140
Unimproved (Ref: Cured)	0.773	0.527 ~ 1.133	0.187	0.808	0.549 ~ 1.190	0.280
Deceased (Ref: Cured)	0.427	0.165 ~ 1.108	0.080	0.543	0.209 ~ 1.409	0.209
Other (Ref: Cured)	0.341	0.273 ~ 1.961	0.535	0.75	0.277 ~ 2.033	0.572
Inter-department transfer	2.119	1.669 ~ 2.691	<0.001	2.209	1.743 ~ 2.801	<0.001
Length of stay (per additional day)	0.572	0.563 ~ 0.581	<0.001	0.554	0.551 ~ 0.557	<0.001
Random effects variance						
σ^2^_u0_(Level 2)	1.163	0.735 ~ 1.842	<0.001^1^	1.668	1.004 ~ 2.770	<0.001^1^
σ^2^_vk_(Level 1)	—	—	—	0.968	0.590 ~ 1.587	<0.001^2^
Log likelihood	−37149.332			−36460.937		

^1^Based on the likelihood ratio test comparing the random-intercept model with the null model (fixed effects only): LR = 18,985.35, df = 1, *p* < 0.001.

^2^Based on the likelihood ratio test comparing the random-coefficient model with the random-intercept model: LR = 1,376.79, df = 1, *p* < 0.001.

## Sensitivity analysis

### Sensitivity analysis excluding category II cases

To verify whether the association between DER and low-value hospitalization was driven by the cost characteristics of Category II (check-up dominated admissions), we re-estimated the core random coefficient model after excluding these cases (After exclusion, *n* = 245,337). Compared with the low-DER reference group, both the normal-DER group (OR = 0.260, 95% CI: 0.184–0.368, *p* < 0.001) and the high-DER group (OR = 0.131, 95% CI: 0.064–0.268, *p* < 0.001) showed significantly lower odds of low-value hospitalization. These findings confirm that the core relationship between DRG-related financial incentives and low-value hospitalization remains robust after excluding Category II cases. The observed association is therefore not merely driven by this specific subgroup, but reflects a genuine underlying link between economic incentives and inappropriate inpatient admission behavior. (Supplementary Table S2).

### Robustness to definitional thresholds

Specifically, we adjusted the cost threshold for Category II admissions—the core criterion for identifying low-value inpatient stays—from the original ≥80 percent to a more lenient ≥75 percent and a stricter ≥85 percent. When using the ≥75 percent threshold, the overall three-year incidence of low-value hospitalizations increased from 4.86 percent to 6.54 percent (an absolute increase of 1.68 percentage points). Conversely, using a stricter threshold of ≥85 percent decreased the overall incidence to 3.35 percent (an absolute decrease of 1.51percentage points, Supplementary Figure S1). Critically, the strong negative association between DER and low-value hospitalization remained virtually unchanged across all thresholds. In all thresholds’ models, cases with High DER continued to have over 90 percent lower odds of being low-value compared to Low DER cases (OR range: 0.023 ~ 0.032), and the variable remained the most significant predictor (*p* < 0.001 in all models). This demonstrates that the identified cost structure is a stable driver of low-value care, independent of minor variations in its measurement (Supplementary Table S3).

## Discussion

This study provides the first quantitative analysis of low-value hospitalizations under China’s DRG reform, revealing a growing source of inefficiency with a clear economic driver. The structure of low-value admissions exhibited substantial fluctuations over the study period, with a pronounced shift from Category III to Category II (Check-up Dominated) cases, which emerged as the dominant subtype by 2024. This upward trend in Category II admissions may be associated with the hospital’s relocation to a new campus in 2022 and the subsequent gradual procurement of large-scale medical equipment, which potentially expanded its capacity for check-up–oriented inpatient services. Causal confirmation is limited by the absence of detailed equipment utilization and patient-level attribution data.

Our core finding—that a lower DRG Medical Expense Ratio (DER) predicts higher risk of low-value admission—exposes a key micro-level incentive. As clarified earlier, DER is calculated as Actual medical expenses of the case divided by Average medical expenses of the corresponding DRG group; a lower DER indicates that the hospital’s actual medical expenses of the case are lower than the DRG group average, creating a stronger economic incentive. This aligns with hospital behavior under DRG’s “no reimbursement for overspending, savings kept” rule: low DER cases offer financial certainty and efficiency, making them attractive to hospitals seeking to avoid cost overruns. We also found notable variation across departments in the strength of the association between low DER and low-value admissions, suggesting differences in internal management strategies and clinical focus among departments. Such heterogeneous responses to financial incentives under fixed payment are widely documented in international prospective payment systems, where provider behavior varies by specialty, case mix, and institutional objectives (26, 27).

These findings are consistent with international research on prospective payment systems. For example, studies on U. S. and European DRG systems have shown that fixed-payment models can be associated with persistent low-value care, even when designed to promote efficiency. In France, as in the United States, DRG incentives can lead hospitals to reduce the provision of high-value services, admit low-cost, low-risk patients with rapid recovery (e.g., minor illnesses), and overprovide low-value services (7, 28). Notably, these studies combine low-value care across both outpatient and inpatient settings. By contrast, our study focuses specifically on high-expense low-value hospitalizations, a more financially impactful component of inappropriate care. This approach extends the literature by examining low-value hospitalization as a unified outcome rather than discrete low-value services. A perspective that is particularly relevant to China’s healthcare system—where tertiary hospitals have strong incentives to maintain high admission volumes. Unlike many international studies, which focus on high-income settings with mature primary care systems, our study highlights the unique challenges of DRG implementation in a middle-income country with a hierarchical medical system still in transition. This distinction underscores our study’s contribution: it provides context-specific evidence for China’s DRG reform, which cannot be directly inferred from international findings.

Furthermore, the regulatory architecture currently possesses gaps that permit such behavior. Audits predominantly focus on coding accuracy and billing compliance rather than on the clinical necessity of the admission itself (29, 30).The absence of a standardized, operational national definition for low-value hospitalization allows institutional interpretations of admission criteria to widen. The significant disparity between UEBMI and URRMI patients (OR = 0.926) highlights how benefit design directly influences utilization. The more generous inpatient reimbursement rates under UEBMI create a powerful financial incentive for patients to seek hospitalization for conditions that could be managed outpatient, effectively transferring economic risk from the patient onto the insurance fund—a clear case of moral hazard in action (31, 32).

To address the incentive distortion identified in our study, two targeted policy implications can be derived from our empirical findings. First, embedding a “Low-Value Care Rule” into provincial DRG platforms could help discourage inappropriate admissions. The identification framework developed in this study can be operationalized as a real-time screening module, flagging claims that meet low-value criteria and triggering tiered payment adjustments or enhanced prior authorization. Second, piloting integrated care pathways for chronic conditions could break the direct link between hospitalization and revenue, encouraging outpatient-based management rather than unnecessary inpatient admissions. These strategies are grounded in our key finding that low DER cases—associated with lower expenses relative to the DRG group average—are strongly predictive of low-value admissions, and they aim to realign institutional incentives with long-term value-oriented goals.

## Limitations and future research

This study has limitations. First, the single-center design may restrict generalizability, as case mix, local DRG implementation details, and hospital management strategies may differ across institutions and regions. Second, the observational design cannot establish a strict causal relationship between DER and low-value hospitalization, even with adjustment for a comprehensive set of confounding variables. In addition, due to model convergence constraints, DRG group fixed effects could not be included, which may leave some residual confounding from unobserved case-mix heterogeneity. Future multi-center studies are warranted to verify the generalizability of these findings. Pre-reform comparative data could also help clarify the causal impact of DRG reform on the emergence of low-value inpatient care.

## Conclusion

China’s DRG reform has successfully introduced a powerful new economic logic into hospital operations. Our analysis demonstrates that without deliberate, corrective design, this logic may produce a specific and profitable type of clinical waste: the low-value hospitalization. The policy imperative is now clear. It is insufficient to merely pay differently; the system must be redesigned to pay smarter and demonstrate value. By integrating value-based signals directly into the payment infrastructure—through intelligent claims screening and strategic pathway contracting, China can steer its historic payment reform toward its ultimate objective: a high-performance healthcare system that rewards the delivery of health, not just the processing of claims. This aligns with the future direction of global health policy: building the value-based payment.

## Data Availability

The data analyzed in this study is subject to the following licenses/restrictions: The data analyzed in this study is subject to the following licenses/restrictions: the health data was anonymously provided by A hospital in city Q; it is not suitable for public disclosure. Requests to access these datasets should be directed to LW, 871618885@qq.com.
