# Supplementary material for: DRG payment, financial signals, and low-value hospitalizations in China
Source: Front Public Health. 2026 May 5;14:1797063. doi: 10.3389/fpubh.2026.1797063 (PMC13183854; doi:10.3389/fpubh.2026.1797063)
Supplement: Supplementary file 1 [file Supplementary_file_1.docx]

Supplementary Materials

Text S1. The Steps of Multilevel mixed-effects logistic regression models.

1. Data collation and variable assignment

The model incorporated patient demographics (age group, gender), insurance type, inter-department transfer, discharge outcome, length of stay, year, and DRG group characteristics. The key explanatory variable was DRG Medical Expense Ratio (DER), defined as the ratio of a case’s actual medical expenses to the average expenses of its DRG group, and categorized as follows: Low DER≤0.4 times, High DER including inpatient total expense≥3 times the average expense of the corresponding DRG (baseline points≤100), ≥2 times (100< baseline points≤300), and≥1.5 times (baseline points >300), and Normal DER (all grouped cases excluding those classified as Low DER and High DER) .

Low (<0.4), Normal (0.4–2.0), or High (>2.0) per local DRG policy. Detailed variable coding is provided in TABLE S1.

TABLE S1 Variable Definitions and Assignment Details

| Indicator | Variable | code |
| --- | --- | --- |
| outcome | low value | non-low value group=0；low value group=1; |
| Level 1 | Year | 2022=1；2023=2；2024=3 |
|  | Gender | male=0; female=1 |
|  | Age group | ≤18=1;18-70=2; ≥70=3 |
|  | Insurance type | UEBMI=1; URRMI=2 |
|  | DER type | low =1; normal =2; high =3 |
|  | Discharge Outcome | Cured=1;Improved=2；Unimproved=3；Deceased=4；other=5 |
|  | Inter-department transfer | No=0; Yes=1 |
| Level 2 | Department | values ranging from 1 to 43 |

2.Model construction

In this model, a binary variable (coded as 1 for low-value hospitalization and 0 otherwise) was defined as the dependent variable. A two-level mixed-effects logistic regression model was employed, where level-1 units were individual patients and level-2 units were clinical departments.^1^

① Null Model (Intercept-Only Model):To determine whether the data TABLE a multilevel structure, a null model without covariates was fitted.

$\mathrm{logit}【P(y_{ij}=1)】=\beta_{0}+u_{0j}$ (Model 1)

Where *yij* is a binary outcome variable (e.g., for low-value hospitalization, *yij* =1 indicates occurrence and *yij =*0 indicates non-occurrence.

[*P* (*yij*​=1) denotes the probability of the outcome occurring for individual *i* in department*j*.

logit(.) represents the log-odds transformation, i.e., logit(*P*)=ln ($\frac{p}{1-p}$).

*β*_0_ is the average intercept; $u_{0j}$is the level-2 random effect (following a normal distribution, $u_{0j}$~*N* (0，$\sigma_{u0}^{2}$), reflecting the deviation of the intercept of group *j*from the overall average intercept;$\sigma_{u0}^{2}$ denotes the variance of the intercepts between groups.

② Random-intercept model

Level-1covariates (year, gender, age, DRG CCR type, etc.) were incorporated, and a random-intercept model was fitted to explore associations with low-value hospitalization.

$\mathrm{logit}【P(y_{ij}=1)】=\beta_{0}+u_{0j}+\sum_{1-k}^{P} \beta_{k}X_{kij}$ (Model 2)

$X_{kij}$ stands for the *k*-th explanatory variable (which can include individual-level or group-level variables); *βk* is the fixed-effect coefficient corresponding to the explanatory variable$X_{kij}$.

③Random-coefficient Model

$logit【P(y_{ij}=1)】=\beta_{0}+u_{0j}+\sum_{1-k}^{P} (\beta_{k}+v_{kj})X_{kij}$ (Model 3)

$v_{kj}$~*N*(0，$\sigma_{vk}^{2}$) denotes the random coefficient corresponding to the explanatory variable $X_{k}$.

Text S2. Sensitivity analysis

Sensitivity Analysis Excluding Category II Cases

TABLE S2 Results of the Sensitivity Analysis Excluding Category II Cases (N=245,337)

| Effects | Random Coefficient Model* | | | |
| --- | --- | --- | --- | --- |
|  | Odds Ratio | Std. Error | *P -*value | 95% Conf. Interval] |
| Fixed Effects |  |  |  |  |
| cons | 0.279 | 0.034 | <0.001 | 0.219~0.354 |
| Insurance type | 0.769 | 0.027 | <0.001 | 0.718~0.823 |
| Age |  |  |  |  |
| 18-70 (Ref: ≤18) | 2.635 | 0.344 | <0.001 | 2.173~3.197 |
| ≥70 (Ref: ≤18) | 3.354 | 0.192 | <0.001 | 2.743~4.102 |
| DRG CCR type |  |  |  |  |
| Normal [Ref: low] | 0.260 | 0.046 | <0.001 | 0.184~0.368 |
| High [Ref: low] | 0.131 | 0.048 | <0.001 | 0.064~0.268 |
| Length of stay | 0.539 | 0.008 | <0.001 | 0.524~0.555 |
| Random Effects Variance | |  |  |  |
| σ^2^_u0_(Level 2) | 1.732 | 0.379 | <0.001 | 1.129~2.670 |
| σ^2^_vk_ (Level1) | 1.655 | 0.375 | <0.001 | 1.062~2.579 |
| Log likelihood | -17842.992 | | | |

*** The results only show** key predictors.

Sensitivity Analyses of defining Category II admissions

We tested three thresholds for defining Category II: ≥75%, ≥80%, ≥85% for the laboratory plus imaging cost ratio. Although the overall incidence of low-value hospitalization varied slightly across thresholds, the strong negative association between DER and low-value admission remained nearly identical in magnitude and significance in all models. These results confirm the stability of the core finding. (As shown in Figure S1 and TABLE S3).


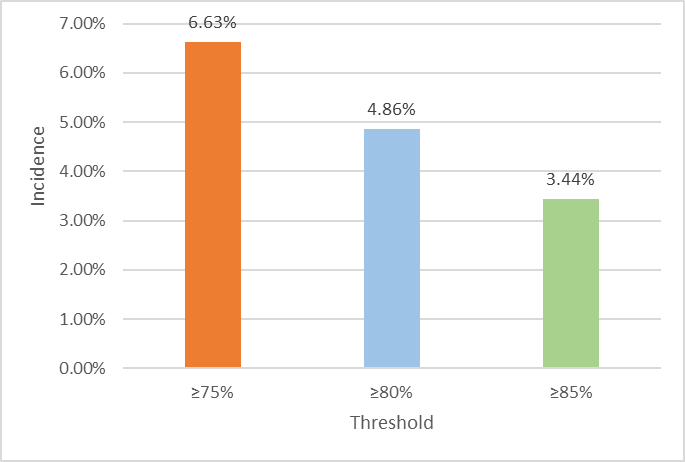
TABLE S3 Model results under the 75% and 85% thresholds

| Effects | ≥75% | | |  | ≥85% | | |
| --- | --- | --- | --- | --- | --- | --- | --- |
|  | Odds Ratio | 95% Confidence Interval | *P -*value |  | Odds Ratio | 95% Confidence Interval | *P -*value |
| Fixed Effects |  |  |  |  |  |  |  |
| cons | 0.252 | 0.181~0.351 | <0.001 |  | 0.094 | 0.069~0.130 | <0.001 |
| Year |  |  |  |  |  |  |  |
| 2023(Ref: 2022) | 0.62 | 0.587~0.655 | <0.001 |  | 0.974 | 0.907~1.046 | 0.466 |
| 2024(Ref: 2022) | 1.476 | 1.322~1.648 | 0.001 |  | 1.553 | 1.327~1.819 | 0.001 |
| Age |  |  |  |  |  |  |  |
| 18-70 (Ref: ≤18) | 0.944 | 0.847~1.053 | 0.300 |  | 1.440 | 1.234~1.680 | <0.001 |
| ≥70 (Ref: ≤18) | 0.794 | 0.706~0.892 | <0.001 |  | 1.355 | 1.153~1.593 | <0.001 |
| DRG CCR type |  |  |  |  |  |  |  |
| Normal [low] | 0.145 | 0.139~0.150 | <0.001 |  | 0.110 | 0.104~0.115 | <0.001 |
| High [low] | 0.028 | 0.024~0.031 | <0.001 |  | 0.027 | 0.226~0.321 | <0.001 |
| Insurance type (Ref: UEBMI) | 1.041 | 1.002~1.081 | 0.039 |  | 0.995 | 0.945~1.048 | 0.852 |
| Inter-department transfer (Ref: No) | 0.375 | 0.314~0.448 | <0.001 |  | 0.348 | 0.263~0.460 | <0.001 |
| Random Effects |  |  |  |  |  |  |  |
| σ^2^_u0_(Level 2) | 1.036 | 0.633~1.697 | <0.001 |  | 0.755 | 0.468~1.216 | <0.001 |
| Log likelihood | -51995.146 | | |  | -30990.231 | | |

Figure S1 The low-value incidence of hospitalizations under three threshold settings

Reference

1. Li Y, Teng D, Shi X, et al. Prevalence of diabetes recorded in mainland China using 2018 diagnostic criteria from the American Diabetes Association: national cross sectional study. Article. *BMJ-BRITISH MEDICAL JOURNAL*. 2020 APR 28 2020;369m997. doi:10.1136/bmj.m997
