# Supplementary material for: DRG payment, financial signals, and low-value hospitalizations in China
Source: Front Public Health. 2026 May 5;14:1797063. doi: 10.3389/fpubh.2026.1797063 (PMC13183854; doi:10.3389/fpubh.2026.1797063)
Supplement: Supplementary file 2 [file Supplementary_file_2.docx]

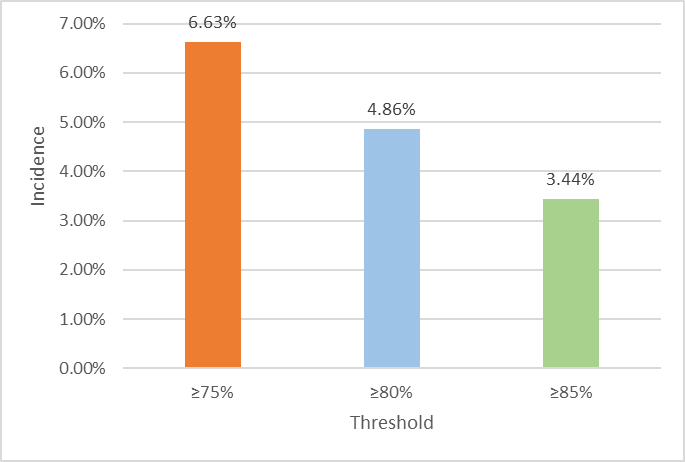


Figure S1 The low-value incidence of hospitalizations under three threshold settings

And below is the raw data table for Figure S1:

| Threshold | Low-Value Cases (n) | Total Admissions | Incidence Rate (%) |
| --- | --- | --- | --- |
| ≥75% | 16,697 | 251,811 | 6.63 |
| ≥80% | 12,248 | 251,811 | 4.86 |
| ≥85% | 8,663 | 251,811 | 3.44 |
