# Supplementary material for: DRG payment, financial signals, and low-value hospitalizations in China
Source: Front Public Health. 2026 May 5;14:1797063. doi: 10.3389/fpubh.2026.1797063 (PMC13183854; doi:10.3389/fpubh.2026.1797063)
Supplement: Supplementary file 3 [file Table_3.docx]

Supplementary TABLE S3

Sensitivity Analyses of defining Category II admissions

We tested three thresholds for defining Category II: ≥75%, ≥80%, ≥85% for the laboratory plus imaging cost ratio. Although the overall incidence of low-value hospitalization varied slightly across thresholds, the strong negative association between DER and low-value admission remained nearly identical in magnitude and significance in all models. These results confirm the stability of the core finding. (As shown in Figure S1 and TABLE S3).

TABLE S3 Model results under the 75% and 85% thresholds

| Effects | ≥75% | | |  | ≥85% | | |
| --- | --- | --- | --- | --- | --- | --- | --- |
|  | Odds Ratio | 95% Confidence Interval | *P -*value |  | Odds Ratio | 95% Confidence Interval | *P -*value |
| Fixed Effects |  |  |  |  |  |  |  |
| cons | 0.252 | 0.181~0.351 | <0.001 |  | 0.094 | 0.069~0.130 | <0.001 |
| Year |  |  |  |  |  |  |  |
| 2023(Ref: 2022) | 0.62 | 0.587~0.655 | <0.001 |  | 0.974 | 0.907~1.046 | 0.466 |
| 2024(Ref: 2022) | 1.476 | 1.322~1.648 | 0.001 |  | 1.553 | 1.327~1.819 | 0.001 |
| Age |  |  |  |  |  |  |  |
| 18-70 (Ref: ≤18) | 0.944 | 0.847~1.053 | 0.300 |  | 1.440 | 1.234~1.680 | <0.001 |
| ≥70 (Ref: ≤18) | 0.794 | 0.706~0.892 | <0.001 |  | 1.355 | 1.153~1.593 | <0.001 |
| DRG CCR type |  |  |  |  |  |  |  |
| Normal [low] | 0.145 | 0.139~0.150 | <0.001 |  | 0.110 | 0.104~0.115 | <0.001 |
| High [low] | 0.028 | 0.024~0.031 | <0.001 |  | 0.027 | 0.226~0.321 | <0.001 |
| Insurance type (Ref: UEBMI) | 1.041 | 1.002~1.081 | 0.039 |  | 0.995 | 0.945~1.048 | 0.852 |
| Inter-department transfer (Ref: No) | 0.375 | 0.314~0.448 | <0.001 |  | 0.348 | 0.263~0.460 | <0.001 |
| Random Effects |  |  |  |  |  |  |  |
| σ^2^_u0_(Level 2) | 1.036 | 0.633~1.697 | <0.001 |  | 0.755 | 0.468~1.216 | <0.001 |
| Log likelihood | -51995.146 | | |  | -30990.231 | | |
